# Exploring genetic variation in insecticide resistance loci in Anopheles gambiae s.l. populations from Ghana

**DOI:** 10.21203/rs.3.rs-11294819/v1

**Published:** 2026-10-23

**Authors:** Anisa Abdulai, Sophie Moss, Kai Brandt-Templeton, Emma Collins, Puck Laan, Isaac Kwame Sraku, Christopher Mfum Owusu-Asenso, Yaw Akuamoah-boateng, Abdul Rahim Mohammed Sabtiu, Fred Aboagye-Antwi, Patrick F. Ayeh-Kumi, Mojca Kristan, Taane G. Clark, Susana Campino, Yaw Asare Afrane

## Abstract

Malaria remains a major public health burden in Sub-Saharan Africa, with Ghana contributing significantly to global cases and deaths. Control relies heavily on vector control interventions, particularly insecticide-treated nets (ITNs) and indoor residual spraying (IRS), but their effectiveness is increasingly threatened by widespread insecticide resistance in malaria vectors. Knowledge of the molecular markers associated with resistance is important for insecticide resistance management in local vector populations. In this study, a low cost, high throughput multiplex amplicon sequencing approach was used for the molecular screening of insecticide resistance alleles in *vgsc* , *rdl* , *ace1* and *gste2* genes, alongside species identification and genetic diversity loci of *An. gambiae* s.l populations from Ghana. A total of 330 *Anopheles gambiae* s.l. from 18 study sites were analysed, including a subset of pyrethroid phenotyped mosquitoes. We detected 31 non-synonymous mutations in the target genes, of which 13 have been previously associated with insecticide resistance (7 in *vgsc* , 2 in *rdl* , 1 in *ace1* , and 3 in *gste2* ). We identified one SNP, T154S in *vgsc* , which was recently detected at a frequency of 24% in *Anopheles* populations from Guinea-Bissau. An additional 17 novel SNPs were identified, including A1553T and L1680F also in the *vgsc* gene. Phylogenetic and genetic analyses of *ITS* 2 and *mitochondrial* sequences showed low nucleotide diversity and limited genetic variation, indicating high gene flow across these *An. gambiae* s.l populations. Overall, this study demonstrates that high-throughput amplicon sequencing provides a cost-effective and scalable method for surveillance of malaria vector populations and can support improved insecticide resistance management and targeted control strategies.

